# Hypothyroidism is Not Associated with Keratoconus Disease: Analysis of 626 Subjects

**DOI:** 10.1155/2019/3268595

**Published:** 2019-10-31

**Authors:** Zsuzsa Flaskó, Elena Zemova, Timo Eppig, László Módis, Achim Langenbucher, Stefan Wagenpfeil, Berthold Seitz, Nóra Szentmáry

**Affiliations:** ^1^Department of Ophthalmology, Saarland University Medical Center, 100 Kirrberger Str., 66421 Homburg, Saarland, Germany; ^2^Department of Ophthalmology, Faculty of Medicine, University of Debrecen, 98 Nagyerdei Str., 4032 Debrecen, Hungary; ^3^Experimental Ophthalmology, Saarland University, 100 Kirrberger Str., 66421 Homburg, Saarland, Germany; ^4^Institute of Medical Biometry, Epidemiology and Medical Informatics, Saarland University Medical Center, 100 Kirrberger Str., 66421 Homburg, Saarland, Germany; ^5^Department of Ophthalmology, Semmelweis University, 39 Mária Str., 1085 Budapest, Hungary

## Abstract

**Purpose:**

To analyze the association between hypothyroidism and keratoconus, we examined blood thyroid hormone levels and corneal tomographic parameters in healthy subjects and patients with keratoconus.

**Methods:**

We included 626 subjects (304 left eyes, 49%; 431 males, 69%; age 38.4 ± 14.3 y). Patients with keratoconus were from our Homburg Keratoconus Center (HKC) (*n* = 463); patients with hypothyroidism were from the Department of Internal Medicine of Saarland Medical University, Homburg/Saar, Germany (*n* = 75); and healthy subjects were from the Department of Ophthalmology of Saarland University Medical Center (*n* = 88). We included only one randomly selected eye of each subject and the first examination data.

**Exclusion criteria:**

Previous thyroid medication, previous ocular surgery, and patients with suspected keratoconus (topographic keratoconus classification, [TKC]: 0 < 1). Patient eyes were classified (TKC) with dedicated, instrument-based, keratoconus detection software provided with the Pentacam. TKC = 0 was considered “normal,” and TKCs ≥ 1 were considered keratoconus. Subjects were also classified as euthyroid or hypothyroid, based on blood thyroid hormone status (i.e., TSH, FT3, and FT4). A multiple logistic linear regression model was constructed to determine the effects of age (covariate), gender, and hypothyroidism (effect sizes) on “TKC-positive” disease.

**Results:**

The significance levels for a constant parameter, sex, thyroid condition, and age were *p* < 0.0001, *p* < 0.0001, *p* < 0.0001, and *p*=0.003, respectively. The odds ratios for age, sex, and hypothyroidism were 0.98, 3.05, and 3.34, respectively. Male sex and a euthyroid condition had significantly positive, clinically relevant effects, and age had a significantly negative, but clinically irrelevant effect on the estimated TKC index.

**Conclusions:**

Keratoconus appeared to occur more often in patients classified as euthyroid than in patients with hypothyroidism. Thus, hypothyroidism alone could not support the development of keratoconus. Based on these results, it should not be mandatory to screen patients with hypothyroidism for keratoconus or patients with keratoconus for hypothyroidism.

## 1. Introduction

Keratoconus is a bilateral, “noninflammatory” condition of the corneal tissue that causes progressive stromal thinning. This condition leads to a cone-shaped, central/paracentral, ectatic cornea. Patients with keratoconus develop irregular astigmatism and myopia [1]. Histopathologically, keratoconus corneas show central or paracentral stromal thinning, ruptures in Bowman's layer, and ring-shaped ferritin depositions at the basal layers of the corneal epithelium (“Fleischer ring”) [2]. Patients with keratoconus can be classified according to severity, based on Scheimpflug technology: 0 (healthy), 1 (suspected), 2 (mild), 3 (moderate), and 4 (severe). Keratoconus can also be classified with keratometric values, as follows: mild (<48 D), moderate (48–54 D), and severe (>54 D) [3].

The prevalence of keratoconus is about 1 : 2000 in the Caucasian population. It commonly appears during puberty, progresses until the third or fourth decade of life, and then, it typically stabilizes [1]. Numerous studies have suggested that hormones, such as estrogen and testosterone, might have an effect on the development of corneal ectasia [4–8].

Keratoconus is a multifactorial disease influenced by environmental and genetic factors [1]. It is considered to be a sporadic disease, but there is also evidence of autosomal dominant [9] and autosomal recessive [10] inheritance. Indeed, studies have shown associations between keratoconus and Down's syndrome (0.5–15%) [11–13]; Turner's syndrome [14]; Leber´s congenital amaurosis [15]; connective tissue disorders, such as Ehlers-Danlos [16] and Marfan [17] syndromes; and mitral valve prolapse. Interleukin-1 may also play a role in the development of keratoconus [18].

The main environmental factor is a high-dose exposure to ultraviolet light (UV). Due to the anterior location of the cornea, about 80% of UVB light, which is entering the eye, is absorbed through the corneal tissue. This type of exposure leads to the local production of reactive oxygen species [2, 19]. In addition, the risk of keratoconus increases with chronic keratocyte apoptosis or ongoing epithelial injury (e.g., due to contact lens wear, chronic eye rubbing, or atopic eye disease) [20, 21].

A thyroid gland dysfunction often results in thyroid-associated ophthalmopathy [22]. The diagnosis of a thyroid gland disorder is based on changes in serum levels of free thyroxine (FT4; normal range: 12–22 pmol/L), free triiodothyronine (FT3; normal range: 3.1–6.8 pmol/L), and thyroid-stimulating hormone (TSH; normal range: 0.27–4.2 mE/L). Hypothyroidism is a pathological condition, where the cells of the human body do not receive the appropriate FT4/FT3 effects. In primary hypothyreosis, FT4 and FT3 are reduced, and TSH is elevated in the blood. Hypothyroidism might also be associated with keratoconus, and it could be one of the causal factors [23–25].

The purpose of the present study was to analyze the association between hypothyroidism and keratoconus, by examining blood thyroid hormone levels, corneal topographic/tomographic parameters, and biomechanical parameters.

## 2. Patients and Methods

This prospective single-center comparative study was conducted at the Department of Ophthalmology, Saarland University Medical Center in Homburg, Germany. The study was approved by the local ethics committee (Number 157/10) and followed the tenets of the Declaration of Helsinki. Informed written consent was obtained from all subjects.

### 2.1. Patient Selection

Patients with keratoconus were enrolled from our Homburg Keratoconus Center (HKC), which was established in 2012 at the Department of Ophthalmology, Saarland Medical University, in Homburg/Saar, Germany (*n* = 463). Patients with hypothyroidism were enrolled from the Department of Internal Medicine of Saarland Medical University, Homburg/Saar, Germany (*n* = 75). Healthy subjects were enrolled from the Department of Ophthalmology of Saarland University Medical Center (*n* = 88). For this study, we acquired the examination data for only one randomly selected eye of each subject.

We acquired data for patients examined between January 2012 and January 2016. We only used the first examination data; follow-up examination data were not used. Exclusion criteria were a previous thyroid medication, previous ocular surgery, and patients with suspected keratoconus (Pentacam topographic keratoconus classification [TKC] 0 < 1). We excluded patients with previous hypothyroid medications to exclude a potential bias of hormone supplementation. Therefore, we only included patients with a new diagnosis of hypothyreosis (which had not yet used medical treatment). Patients with previous ocular surgery were excluded, because surgery could influence corneal topography. Patients with suspected keratoconus were excluded, because a false diagnosis of keratoconus could also result in a bias in the group of patients with keratoconus.

This study included 4 patient groups: (1) subjects classified as euthyroid without keratoconus (controls), (2) patients classified as euthyroid with keratoconus, (3) patients with hypothyroidism, but without keratoconus, and (4) patients with hypothyroidism and keratoconus. Patient eyes were classified as keratoconus with dedicated, instrument-based, keratoconus detection software (topographic keratoconus classification, TKC) provided with the Pentacam (see below). A TKC of 0 was considered normal, and TKC values of 1 or higher were considered keratoconus. However, as mentioned above, patients with suspected keratoconus (TKC 0 < 1) were excluded from the study.

Patients were considered euthyroid or hypothyroid, based on blood thyroid hormone levels. Normal values were defined as follows: 12–22 pmol/L for FT4, 3.1–6.8 pmol/L for FT3, and 0.27–4.2 mE/L for TSH. Patients with hormone levels within these ranges were classified as euthyroid. Patients with lower FT4 and FT3 values or higher TSH values were classified as patients with hypothyroidism [22].

### 2.2. Examination Protocol

All subjects received a complete standard ophthalmological examination. In addition, subjects were examined with a topographic modeling system for corneal topography (TMS-5; Tomey, Nürnberg, Germany) and with a rotating Scheimpflug camera (Pentacam HR, Oculus Optikgeräte GmbH, Wetzlar, Germany). From the anterior corneal surface data, we extracted the following parameters: the surface asymmetry index (SAI), surface regularity index (SRI), Klyce/Maeda keratoconus index (KCI), Smolek/Klyce keratoconus severity index (KSI), and keratoconus prediction index (KPI). From the tomographic examination, the following parameters were extracted: the index of surface variance (ISV), index of surface asymmetry (IVA), keratoconus index (KI), center keratoconus index (CKI), index of height asymmetry (IHA), and index of height decentration (IHD). In addition, subjects were examined with the ocular response analyzer (ORA, Reichert Ophthalmic Instruments, Buffalo, NY) to measure the biomechanical properties of the cornea, including the following parameters: keratoconus match index (KMI), corneal hysteresis (CH), and corneal resistance factor (CRF). We used the noncontact EM-3000 specular microscope (Tomey, Nürnberg, Germany) for evaluating the endothelial cell density (ECD) and central corneal thickness.

This study included 626 subjects (304 left eyes, 49%; 431 males, 69%; age 38.4 ± 14.3 y). Subjects were classified as euthyroid (*n* = 458, 73%) or hypothyroid (*n* = 168, 27%), based on blood thyroid hormone status (TSH, FT3, FT4). Of the 458 subjects classified as euthyroid, 375 (80%) had keratoconus and 83 (20%) did not have keratoconus. Of the total study population of 626 subjects, 465 (74%) had keratoconus, according to the TKC (Pentacam), and of these, 90 (19%) had hypothyroidism. Of the 161 patients without keratoconus, 78 (48%) had hypothyroidism. Of the controls, 5 were diagnosed with a thyroid disorder, and none had keratoconus.

### 2.3. Statistical Analysis

We performed statistical analyses with SPSS 21.0 software. The normality distribution was checked for all parameters with the Kolmogorov–Smirnov test.

The TMS-5, Pentacam, ORA, and EM-3000 data were analyzed descriptively, and results are expressed as the mean and standard deviation or the median and interquartile range for the 4 study groups. A nonparametric Mann–Whitney *U*-test was used to evaluate differences in parameters between subjects classified as euthyroid, without or with keratoconus (patient groups 1 and 2); between subjects with hypothyroidism, without or with keratoconus (patient groups 3 and 4); between subjects classified as euthyroid or hypothyroid without keratoconus (patient groups 1 and 3); and between subjects classified as euthyroid or hypothyroid with keratoconus (patient groups 2 and 4).

A multiple logistic linear regression model was constructed to determine the effects of age (covariate), gender (dichotomous factor), and hypothyroidism (dichotomous factor: 1 = hypothyroidism, 0 = euthyroidism) on TKC-positive disease (dichotomous factor: 0 = no keratoconus [TKC = 0], 1 = keratoconus [TKC = 1–4]). This model was tested with a receiver-operating characteristics (ROC) analysis, and the area under curve (AUC) was extracted as a general descriptor. The maximum Youden index value was used as the threshold for separating keratoconus from nonkeratoconus.

*p* values below 0.05 were considered statistically significant.

## 3. Results

**Figure 1:**
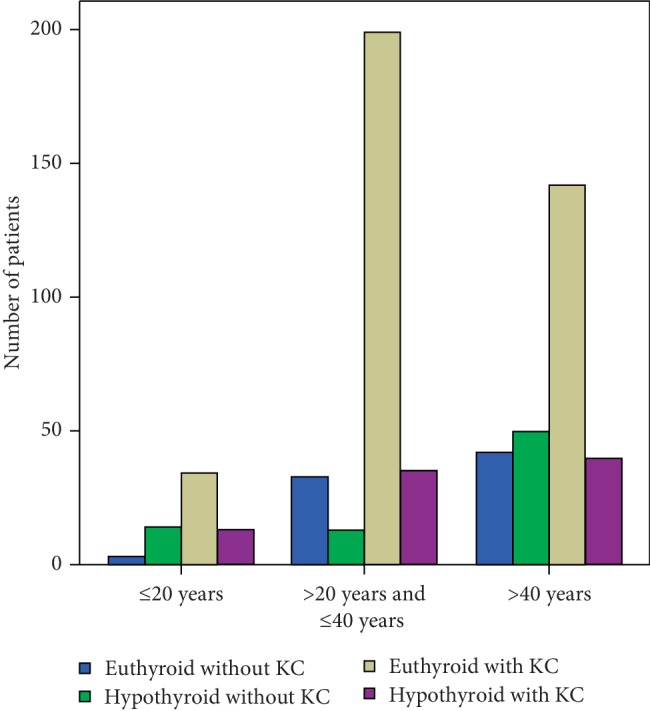
The disease distribution in different age groups.

Figure 1 shows the disease distribution in different age groups.  Table 1 displays the serum FT4, FT3, and TSH levels in different disease groups.  Table 2 shows results from the TMS-5 analysis of the anterior corneal surface.  Table 3 shows the results from Pentacam corneal tomography.  Table 4 shows the corneal biomechanical properties from the ORA evaluation.  Table 5 shows the results from the corneal endothelium evaluation with the EM-3000 specular endothelial microscope.

We found significant differences between disease groups in all corneal topographic, tomographic, and biomechanical parameters. In addition, the central corneal thickness, measured with the EM-3000, was significantly different between subjects classified as euthyroid, without or with keratoconus, and between subjects with hypothyroidism, without or with keratoconus (*p* < 0.05 for all; Tables 2–5). In addition, subjects classified as euthyroid or hypothyroid without keratoconus showed significant differences in the SRI, measured with the TMS-5, the ISV, the IVA, and the IHD, measured with the Pentacam, and the KMI, measured with the ORA (*p* < 0.05 for all; Tables 2–4). The endothelial cell density was also significantly different between subjects classified as euthyroid, without or with keratoconus (*p* < 0.05; Table 5).

In the generalized linear model, the absence/presence of TKC (0 = no keratoconus, 1 = keratoconus) was modeled with the following effect sizes: gender (1 = male, 2 = female) and hypothyroidism (0 = euthyroid, 1 = hypothyroid). Additionally, age (in years) was included as a covariate.

The model equation yielded the TKC estimation, as follows:

(1) $$\begin{array}{c} {\text{TKC}}_{\text{est}}=0.557\;+\;\langle \begin{array}{c} 0.218\left(\text{if male}\right)\\ 0\left(\text{if female}\right) \end{array}\rangle \;+\;\langle \begin{array}{c} 0.231\left(\text{if euthyroid}\right)\\ 0\left(\text{if hypothyroid}\right) \end{array}\rangle -0.003\cdot \text{age}. \end{array}$$

The significance levels for the constant term, sex, thyroid condition, and age were *p* < 0.0001, *p* < 0.0001, *p* < 0.0001, and *p*=0.003, respectively. The odds ratios for age, sex, and hypothyroidism were 0.98, 3.05, and 3.34, respectively. Based on the linear model, male sex and the euthyroid condition had clinically relevant, significantly positive effects on the estimated TKC index. In addition, age had a tiny, significantly negative, but clinically irrelevant, effect on the estimated TKC index.

**Figure 2:**
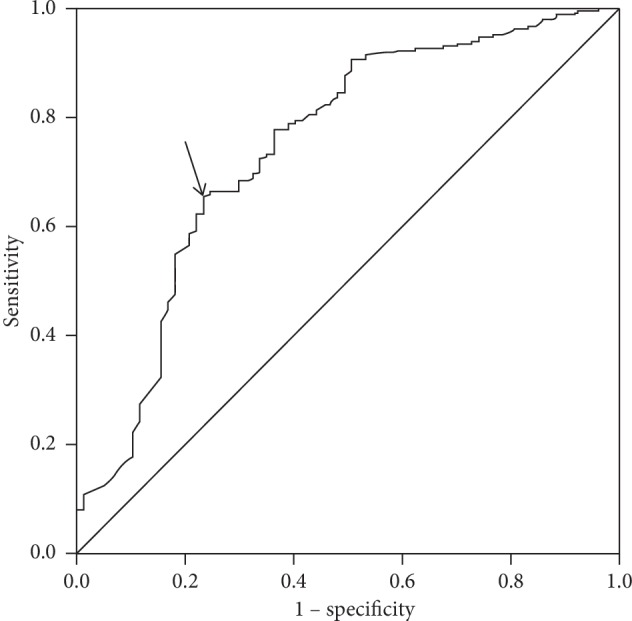
Receiver-operating characteristics (ROC) curve for estimating the TKC. A linear estimation equation was used to determine the best threshold for distinguishing between keratoconus and normal eyes with the ROC curve. The area under the ROC curve is 0.74 (arrow). The maximum Youden index is marked with an arrow on the graph. The Youden index is (sensitivity + specificity − 1) 0.422.

As proof-of-concept, we used this linear TKC estimation equation to determine the best threshold for distinguishing between keratoconus and normal corneas, with an ROC analysis. The ROC curve had an AUC of 0.74 (Figure 2). The maximum Youden index was 0.422, and the respective threshold was 0.74. The analysis yielded a sensitivity of 0.656 and a specificity of 0.766. The mean absolute estimation error was 0.210 ± 0.241.

## 4. Discussion

The major finding of our study was that patients classified as euthyroid appeared to have keratoconus more often than patients with hypothyroidism. As commonly described, KC was more often present in males than in females. Moreover, the risk of developing keratoconus decreased by 2% with each year of age (odds ratio: 0.98).

Hormonal influences on the cornea have interested researchers for a long time. During embryonic life, thyroid hormone is essential for the development of corneal transparency, and it is an important factor in initiating corneal dehydration [26, 27]. The presence of thyroxine receptors was verified in chicken [28] and human corneas [24]. Furthermore, a certain quantity of thyroxine is necessary for the synthesis of corneal collagen [29].

During childhood, serum thyroxine (T4) levels decrease in concert with a progressive reduction in thyroxine-binding globulin (TBG). TBG levels are lowest in puberty, when keratoconus typically appears or progresses [30]. The thyroxine-binding capacity of TBG is also influenced by gender hormones: estrogen increases and testosterone decreases TBG binding capacity [31]. Interestingly, in spontaneous mutant keratoconus mice, keratoconus was observed almost exclusively in mature males, but not in castrated animals [4]. In spontaneous mutant keratoconus mice, systemic testosterone also led to keratoconus in females.

Estrogen receptors were also identified in human corneas [8, 32]. One report hypothesized that keratoconus might be triggered by estrogen [8], which might potentially explain the keratectasia observed after refractive surgery during pregnancy [5, 33]. The pregnancy-induced progression of keratoconus was also studied by Bilgihan et al. [34]. Fink et al. investigated the influence of gender and hormone status on the severity and progression of keratoconus. They could not verify any significant differences between midlife males, “hormone-active” females, and “hormone-inactive” females [6].

Kahán et al. analyzed tear and blood samples of patients with keratoconus. One-third of those patients had hypothyroidism, half were classified as euthyroid, and all patients with both hyperthyroidism and keratoconus were males [35]. In comparison, in our study, we found that approximately 80% of patients classified as euthyroid and 20% of patients with hypothyroidism had keratoconus, after excluding data from patients with hyperthyroidism. One previous study claimed that keratoconus appeared after a thyroidectomy [36]. Others found a reversible increase in corneal thickness in patients with hypothyroidism, which decreased with medical treatment [37].

The association between hypothyroidism and keratoconus is rare. However, this association might be found sporadically or in specific environmental or clinical conditions, such as pregnancy or allergy [38]. The relationship between thyroid gland dysfunction and keratoconus progression was described as hypothyroxinemia in one patient during pregnancy, by Gatzioufas and Thanos [23]. They found that *K*_max_ and corneal thickness values of this patient normalized after giving supplementary thyroxine medication [23]. Similar results were found in a study by Hoogewound et al. [39]. In addition, Hafezi and Iseli found that iatrogenic keratectasia progressed in pregnancy, despite the administration of crosslinking therapy [40]. More recently, Thanos et al. described a crucial role of T4 in keratoconus pathophysiology [25]. They found a 13.6% prevalence of thyroid gland dysfunction among patients with keratoconus, which was higher than the prevalence in the general population (∼2%) [25].

Our findings that patients classified as euthyroid appeared to have keratoconus more often than patients with hypothyroidism contradicted results found in previous studies. However, with the exception of the study by Thanos et al., all other clinical studies mentioned above were case reports, which did not include data for a large patient population. In addition, no previous study analyzed the effect of age or gender on disease characteristics, which we analyzed in the present study. We excluded biases due to different patient ages or genders with our multiple logistic linear regression model.

It was not surprising to find significant differences in all topographic, tomographic, and ORA parameters between patients classified as euthyroid with/without keratoconus and between patients with hypothyroidism with/without keratoconus [3]. For example, we found significant differences in SRIs (TMS-5), ISVs, IVAs, and IHDs (Pentacam) between patients classified as euthyroid or hypothyroid, in the absence of keratoconus. In patients with hypothyroidism, these findings could be due to dry eye disease, which was not analyzed in the present study. Dry eye disease is part of thyroid-induced ophthalmopathy [22]. Moreover, dry eye disease is known to cause changes in topographic and tomographic parameters [41].

We found an increase in the KMI, measured with ORA, among patients with hypothyroidism compared to patients classified as euthyroid, in the absence of keratoconus. This finding was partly consistent with the study by Gatzioufas et al. [24]. They showed that ORA parameters might increase in patients with hypothyroidism, compared to normal controls. They found increases in the CH and CRF, measured with ORA, among patients with hypothyroidism; however, unlike our findings, they did not detect a difference in KMI between groups.

## 5. Conclusion

Keratoconus appeared to occur more often in subjects classified as euthyroid compared to subjects with hypothyroidism. Thus, our findings showed that hypothyroidism alone could not support the etiology of keratoconus. Nevertheless, in rare cases, hypothyroidism has been reported in the pathogenesis of keratoconus; thus, it represents a risk factor during pregnancy and in rare conditions, particularly in males, due to the high levels of testosterone and dehydroepiandrosterone sulphate. Therefore, hypothyroidism could be considered an adjunctive progression factor, but not an etiologic factor [4–8]. Although these data require further analysis, the present results suggest that it should not be mandatory to screen patients with hypothyroidism for keratoconus or patients with keratoconus for hypothyroidism.

## Figures and Tables

**Table 1:** Serum thyroid hormones: FT4 (pmol/L), FT3 (pmol/L), and TSH (mE/L) measured in different disease groups.

Disease group	FT4	FT3	TSH
1. Euthyroid without keratoconus (*n* = 83)	1.20 ± 0.14	3.32 ± 0.42	1.50 ± 0.62
	1.18; 0.22	3.30; 0.60	1.48; 0.83

2. Euthyroid with keratoconus (*n* = 375)	1.20 ± 0.13	3.45 ± 0.45	1.64 ± 0.57
	1.19; 0.21	3.4; 0.50	1.63; 0.85

3. Hypothyroid without keratoconus (*n* = 69)	1.38 ± 0.27	3.11 ± 0.47	2.17 ± 1.74
	1.36; 0.34	3.00; 0.70^a^	1.67; 2.43

4. Hypothyroid with keratoconus (*n* = 86)	1.29 ± 0.21	3.41 ± 0.46	2.72 ± 1.48
	1.25; 0.24	3.40; 0.50	3.10; 2.20

Values represent the mean ± standard deviation or the median; interquartile range, as indicated. ^a^For this group, *n* = 65.

**Table 2:** Anterior corneal surface parameters measured with the TMS-5 in different disease groups.

Disease group	SAI	SRI	KCI	KSI	KPI
1. Euthyroid without keratoconus (*n* = 75)	0.55 ± 0.36	0.30 ± 0.36	2.29 ± 6.43	1.79 ± 5.76	0.21 ± 0.02
	0.44; 0.37	0.19; 0.35^a^	0.00; 0.00	0.00; 0.00	0.21; 0.03

2. Euthyroid with keratoconus (*n* = 341)	2.57 ± 1.41	1.08 ± 0.62	64.08 ± 34.38	53.83 ± 25.97	0.36 ± 0.11
	2.52; 1.86^b^	1.01; 0.93	76.80; 58.50	56.90; 39.90	0.36; 0.16^b^

3. Hypothyroid without keratoconus (*n* = 57)	0.47 ± 0.31	0.17 ± 0.23	4.76 ± 10.73	2.72 ± 8.26	0.21 ± 0.02
	0.39; 0.33	0.07; 0.19	0.00; 0.00	0.00; 0.00	0.21; 0.03

4. Hypothyroid with keratoconus (*n* = 68)	2.55 ± 1.42	1.15 ± 0.62	60.62 ± 34.71	57.46 ± 27.21	0.35 ± 0.34
	2.39; 1.97	1.06; 0.89	69.65; 60.80	61.35; 41.85	0.34; 0.17

Difference between groups					
1-2	*∗∗*	*∗∗*	*∗∗*	*∗∗*	*∗∗*
3-4	*∗∗*	*∗∗*	*∗∗*	*∗∗*	*∗∗*
1–3	—	*∗∗*	—	—	—
2–4	—	—	—	—	—

Values represent the mean ± standard deviation or the median; interquartile range, as indicated. ^a^For this group, *n* = 74; ^b^for this group, *n* = 340; ^*∗*^*p* < 0.05; *∗∗p* < 0.01. SAI: surface asymmetry index; SRI: surface regularity index; KCI: Klyce/Maeda keratoconus index; KSI: Smolek/Klyce keratoconus severity index; KPI: keratoconus prediction index.

**Table 3:** Corneal tomographic parameters measured with the Pentacam in different disease groups.

Disease group	ISV	IVA	KI	CKI	IHA	IHD
1. Euthyroid without keratoconus (*n* = 82)	21.31 ± 10.98	0.18 ± 0.15	1.02 ± 0.03	1.07 ± 0.67	6.42 ± 5.59	0.02 ± 0.10
	18.00; 14.00	0.15; 0.14	1.03; 0.04	1.00; 0.01	5.20; 6.60	0.01; 0.01

2. Euthyroid with keratoconus (*n* = 374)	101.17 ± 50.28	1.07 ± 0.52	1.26 ± 0.18	1.06 ± 0.24	34.65 ± 57.29	0.13 ± 0.11
	93.00; 59.00	1.00; 0.72	1.23; 0.19	1.04; 0.09	24.40; 33.42	0.11; 0.09

3. Hypothyroid without keratoconus (*n* = 78)	16.20 ± 6.25	0.12 ± 0.07	1.02 ± 0.02	1.00 ± 0.007	4.92 ± 4.46	0.01 ± 0.009
	15.00; 7.00	0.11; 0.08	1.02; 0.02	1.00; 0.01	3.40; 5.85	0.008; 0.008

4. Hypothyroid with keratoconus (*n* = 90)	102.64 ± 61.11	1.00 ± 0.49	1.25 ± 0.17	1.06 ± 0.08	30.87 ± 29.2	0.12 ± 0.07
	94.00; 66.75	0.98; 0.65	1.23; 0.22	1.05; 0.08	23.60; 29.17	0.11; 0.10

Difference between groups:						
1-2	*∗∗*	*∗∗*	*∗∗*	*∗∗*	*∗∗*	*∗∗*
3-4	*∗∗*	*∗∗*	*∗∗*	*∗∗*	*∗∗*	*∗∗*
1–3	*∗∗*	*∗∗*	—	—	—	*∗∗*
2–4	—	—	—	—	—	—

Values represent the mean ± standard deviation or the median; interquartile range, as indicated. ^*∗*^*p* < 0.05; *∗∗p* < 0.01. ISV: index of surface variance; IVA: index of surface asymmetry; KI: keratoconus index; CKI: center keratoconus index; IHA: index of height asymmetry; IHD: index of height decentration.

**Table 4:** Biomechanical parameters measured with the ocular response analyzer in different disease groups.

Disease group	KMI	CH	CRF
1. Euthyroid without keratoconus (*n* = 78)	0.71 ± 0.39	10.00 ± 1.90	9.89 ± 1.95
	0.67; 0.53	9.95; 2.62	9.55; 2.85

2. Euthyroid with keratoconus (*n* = 325)	0.09 ± 0.40	8.15 ± 1.73	7.06 ± 2.08
	0.06; 0.57	8.10; 2.20	6.90; 2.50

3. Hypothyroid without keratoconus (*n* = 72)	0.81 ± 0.32	10.28 ± 1.88	10.00 ± 1.96
	0.88; 0.42	9.95; 2.62	9.60; 2.80

4. Hypothyroid with keratoconus (*n* = 77)	0.04 ± 0.41	8.18 ± 2.05	7.00 ± 2.46
	0.02; 0.57	8.30; 2.55	7.10; 2.70

Difference between groups:			
1-2	*∗∗*	*∗∗*	*∗∗*
3-4	*∗∗*	*∗∗*	*∗∗*
1–3	*∗*	—	—
2–4	—	—	—

Values represent the mean ± standard deviation or the median; interquartile range, as indicated. ^*∗*^*p* < 0.05; *∗∗p* < 0.01. KMI: keratoconus match index; CH: corneal hysteresis; CRF: corneal resistance factor.

**Table 5:** Corneal endothelium evaluated with the EM-3000 specular microscope in different disease groups.

Disease group	Endothelial cell density	Central corneal thickness
1. Euthyroid without keratoconus	2606 ± 360	507.69 ± 99.49
	2690; 452	525.00; 60
	(*n* = 70)	(n = 65)

2. Euthyroid with keratoconus	2523 ± 362	451.44 ± 107.99
	2558; 421	474.00; 73
	(*n* = 302)	(*n* = 277)

3. Hypothyroid without keratoconus	2586 ± 387	512.26 ± 98.75
	2598; 401	528.00; 41
	(*n* = 68)	(n = 65)

4. Hypothyroid with keratoconus	2501 ± 382	459.36 ± 100.33
	2558; 381	479.00; 83
	(*n* = 62)	(*n* = 58)

Difference between groups		
1-2	*∗*	*∗∗*
3-4	—	*∗∗*
1–3	—	—
2–4	—	—

Values represent the mean ± standard deviation or the median; interquartile range, as indicated. ^*∗*^*p* < 0.05; *∗∗p* < 0.01.

## Data Availability

The data used to support the findings of this study are included within the article.
